# Good Manufacturing Practice-Derived Human Liver Stem Cell Extracellular Vesicles Attenuate Liver Fibrosis In Vivo

**DOI:** 10.3390/cells15080661

**Published:** 2026-04-09

**Authors:** Elena Ceccotti, Veronica Dimuccio, Chiara Pasquino, Massimo Cedrino, Maria Beatriz Herrera Sanchez, Cristina Grange, Federico Figliolini, Giorgio Nicolò, Federica Antico, Selene Limoncelli, Giulio Mengozzi, Giulia Gioiello, Marta Tapparo, Fabio Cattelino, Renato Romagnoli, Giovanni Camussi, Valentina Fonsato, Stefania Bruno

**Affiliations:** 1 Department of Medical Sciences, University of Torino, 10126 Torino, Italy; elena.ceccotti@unito.it (E.C.); cristina.grange@unito.it (C.G.); giulio.mengozzi@unito.it (G.M.); marta.tapparo@gmail.com (M.T.); giovanni.camussi@unito.it (G.C.); 2 Cell Factory (CLF), University of Torino, 10126 Torino, Italy; veronica.dimuccio@unito.it (V.D.); chiara.pasquino@unito.it (C.P.); federico.figliolini@gmail.com (F.F.); giorgio.nicolo@unito.it (G.N.); valentina.fonsato@unito.it (V.F.); 3 Department of Molecular Biotechnology and Health Science, University of Torino, 10126 Torino, Italy; federica.antico@unito.it; 4 Molecular Biotechnology Center Guido Tarone, University of Torino, 10126 Torino, Italy; massimo.cedrino@unito.it (M.C.); fabio.cattellino92@gmail.com (F.C.); 5 2i3T, Incubatore per le Imprese, University of Torino, 10126 Torino, Italy; mariabeatriz.herrera@unito.it; 6 Clinical Biochemistry Laboratory, Città della Salute e della Scienza, 10126 Torino, Italy; slimoncelli@cittadellasalute.to.it (S.L.); giulia.gioiello@unito.it (G.G.); 7 General Surgery 2U-Liver Transplant Center, Department of Surgical Sciences, AOU Città della Salute e della Scienza di Torino, 10126 Torino, Italy; renato.romagnoli@unito.it

**Keywords:** GMP conditions, thioacetamide, chronic liver disease, HLSCs

## Abstract

**Highlights:**

**What are the main findings?**

- It is feasible to produce HLSC-EVs under fully GMP-compliant conditions using a standardized and scalable manufacturing workflow.
- GMP-grade EVs retained antifibrotic activity both in vitro and in vivo.

**What are the implications of the main findings?**

- The standardized workflow ensures consistent physicochemical and biological properties that support their development as a clinical-grade biological product.
- The in vitro and in vivo antifibrotic effect indicate that the transition from research-grade isolation procedures to GMP-compatible production does not compromise their therapeutic potential.

**Abstract:**

Human liver stem cells (HLSCs) are a mesenchymal stromal cell (MSC)-like population isolated from adult liver biopsies. HLSCs share key characteristics with MSCs, including phenotype and differentiation capabilities. Previous studies have demonstrated that HLSCs promote regeneration in different experimental models of acute and chronic tissue injury and that HLSC-derived extracellular vesicles (HLSC-EVs) recapitulate the therapeutic effects of the cells of origin. This study aimed to determine whether HLSC-EVs, obtained and characterized under good manufacturing practice (GMP) conditions, can influence the progression of liver fibrosis in vivo. The EV production process was carried out under GMP conditions to generate batches of HLSC-EVs by tangential flow filtration. To assess their therapeutic potential, an in vivo model of hepatic fibrosis was established through administration of thioacetamide (TAA). In TAA-treated mice, EV administrations attenuated fibrosis progression. Molecular analyses showed a significant reduction in the expression levels of key pro-fibrotic genes. At the functional level, EV administration resulted in a significant reduction in plasma alanine aminotransferase levels and an increase in albumin levels, indicating improved liver function. These data indicate that HLSC-EVs, produced under GMP conditions, display antifibrotic effects in a chronic liver disease model, leading to improved liver function and histology.

## 1. Introduction

Liver fibrosis occurs as a wound-healing response to chronic liver insults, such as alcohol consumption, metabolic dysfunction-associated steatotic liver disease, genetic disorders, viral infections, autoimmune diseases, and cholestatic diseases. During fibrosis development, normal hepatic tissue is progressively substituted with fibrotic tissue, formed by the excessive accumulation of extracellular matrix (ECM) proteins, particularly collagen [1]. Fibrosis compromises liver functions, leading to significant clinical complications and may progress to cirrhosis and hepatocellular carcinoma [2]. A growing number of experimental studies have indicated that extracellular vesicles (EVs), derived from different sources, may represent an innovative and promising option to counteract the progression of liver fibrosis [3,4,5].

EVs are a heterogeneous population of membrane-delimited particles released by all cell types. EVs function both through direct signaling by receptor interaction and through the delivery of their molecular cargo, usually composed of different RNA types, proteins, and bioactive lipids [6,7]. For a clinical translation, it is necessary to ensure EV safety and efficacy. This includes the development of clinical-grade EVs under stringent manufacturing processes that comply with good manufacturing practices (GMPs) and implement quality assurance protocols [8,9].

Among various EV sources, human liver stem cells (HLSCs) represent a promising candidate for the treatment of hepatic fibrosis. HLSCs are a mesenchymal stromal cell (MSC)-like population isolated from adult liver biopsies, which express typical MSC surface antigens and possess immunomodulatory and differentiation capacities, similar to MSCs [10]. Previous experimental studies have demonstrated that HLSCs promote recovery in different experimental models of acute and chronic tissue injury and restore hepatic function in genetic liver disease [10]. Moreover, in a Phase I clinical trial, the percutaneous intrahepatic administration of HLSCs was safe and well-tolerated in three infants with inherited neonatal-onset hyperammonemia caused by different genetic diseases (argininosuccinic aciduria and methylmalonic acidemia). No local or systemic adverse events were reported, and no immunosuppressive regimen was applied in these patients. All patients remained clinically stable, and metabolic decompensations were not evident after HLSC administration [11].

As reported in different experimental settings, HLSC-derived EVs (HLSC-EVs) are able to mimic the therapeutic effects of their parental cells. HLSC-EVs, when produced using research-grade isolation method such as ultracentrifugation, showed an antifibrotic effect in hepatic models, both in vitro and in vivo [12,13]. However, for clinical translation, EVs must be manufactured using standardized and scalable processes that comply with GMP requirements. Importantly, modifications in production methods and purification strategies may affect EV biological activity. Therefore, it remains essential to verify whether EVs produced under fully GMP-compliant conditions retain therapeutic efficacy previously observed with research-grade preparations.

The present study aimed to evaluate the therapeutic efficacy of HLSC-EVs, obtained under GMP conditions, in mice with thioacetamide (TAA)-induced liver fibrosis, a widely recognized model of chronic liver injury [14,15,16].

The antifibrotic effect of EVs was assessed by evaluating their impact on hepatic function, histology, and gene expression of key fibrosis markers. Additionally, the antifibrotic effect of EVs was also investigated in vitro, demonstrating the capacity to attenuate the activated state of fibroblasts.

## 2. Materials and Methods

### 2.1. EV Production

The EV production process was carried out under GMP conditions to generate batches of HLSC-EVs. These batches consisted of multiple cryobags, each containing a concentration of 4 × 10^9^ EVs/mL, resuspended in 50 mL of freezing solution.

The production process involved sequential steps as follows:

1. HLSC stock expansion: starting from a certified master cell bank (MCB), HLSCs were expanded to produce and collect the conditioned medium (CM);
2. HLSC-EV isolation and formulation: EVs were isolated by tangential flow filtration (TFF), followed by filtration and final drug product formulation.

### 2.2. HLSC Culture

HLSCs were obtained and cultured in GMP conditions starting from a certified MCB, obtained from a single donor, and generated by the Officina Farmaceutica “University of Torino” (Turin, Italy). The cell manipulation was performed in grade A biological safety cabinets with a surrounding grade B environment, according to the EudraLex [17]. In every working section, viable and non-viable particles were continuously monitored, along with differential pressure, surface, and operators.

The liver fragment was enzymatically digested with 0.6 mg/mL GMP-grade collagenase I (Nordmark Pharma GmbH, Uetersen, Germany) and 0.73 mg/mL GMP-grade neutral protease (Nordmark Pharma) for 15 min at 37 °C, followed by a filtration step through a 40 mm filter. Cells were seeded in culture (4 × 10^4^ cell/cm^2^) in T75 flasks in the presence of expansion medium composed of α-MEM (Lonza, Verviers, Belgium) supplemented with 10% fetal bovine serum (FBS, Gibco, Grand Island, NY, USA), 4 ng/mL human GMP recombinant epidermal growth factor (EGF, Miltenyi Biotec, Bergisch Gladbach, Germany), 4 ng/mL human GMP recombinant basic fibroblast growth factor (FGFb, Miltenyi Biotec), and maintained in a humidified incubator with 5% CO_2_ at 37 °C. After about 10 days of culture, cells were detached and seeded at a density of 3–5 × 10^3^ cells/cm^2^ (in T175) for expansion. After two expansions, cells were harvested and frozen in vials containing 5 × 10^6^ cells, in the presence of FBS and 10% dimethyl sulfoxide (DMSO, Alchimia, Ponte San Nicolò, Italy) using a controlled rate freezer (CRF), to generate the MCB, stored at a controlled temperature of <−140 °C.

A cell stock (CS) was created starting from two vials of MCB cultured in two hyperflasks (HFs, Corning, NY, USA). When 90% confluency was reached, cells were collected by harvesting and frozen through a CRF in vials containing 5 × 10^6^ cells/vial and stored in vapor of liquid nitrogen.

Two vials of CS were thawed, cultured in HFs, and expanded into a further 16 HFs. When cells reached 85–90% confluency, they were washed with Dulbecco’s Phosphate-Buffered Saline (DPBS, Lonza Verviers, Belgium) and then cultured in basal medium (α-MEM). After 18 ± 4 h, the conditioned medium (CM) was collected and immediately subjected to the TFF process.

All critical raw materials used in the manufacturing of MCB and CS were chemically defined or GMP-grade and approved in accordance with specific acceptance criteria, especially for bovine serum HI&GI selected according to the guideline for the manufacture of human biological medicinal products EMA/CHMP/BWP/457920/2012.

### 2.3. Tangential Flow Filtration (TFF) Procedure

**Figure 1:**
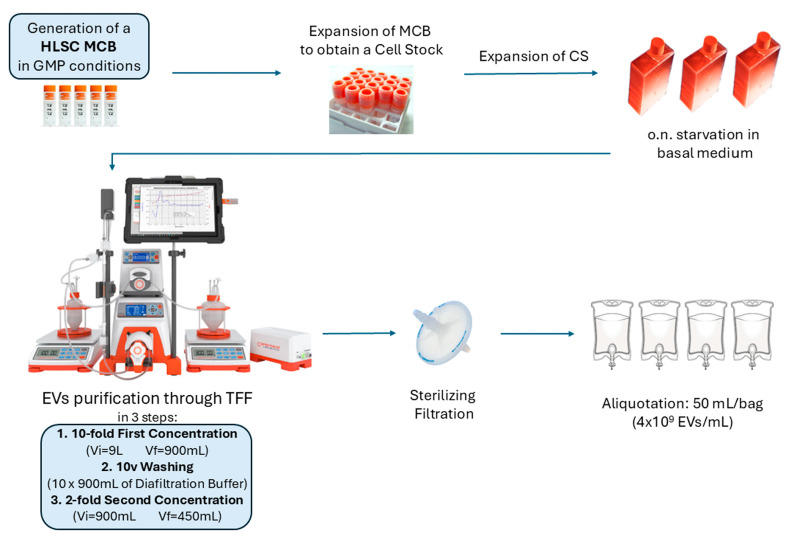
Schematic representation of the GMP production process leading to the purification of EVs from the supernatant of HLSCs.

A KrosFlo^®^2i TFF System (Spectrum Labs, Los Angeles, CA, USA) and the corresponding 21 CFR compliant software (KF Comm 2C) (Repligen, Waltham, MA, USA) were used to concentrate and purify EVs through TFF (Figure 1). Conditioned medium was first filtered through a Supor EAV membrane in mini Kleenpak capsules, 0.2 µm (Pall Corporation, Port Washington, NY, USA) for bioburden and cell debris removal. TFF was performed with a 100 kDa cut-off filter module (N04-E100-10-N) (Spectrum Labs): it was first washed with 6.5 L of sterile water, and then the whole flowpath was primed with 1.5 L of a-MEM. As the TFF system was closed, EV isolation was performed under grade C conditions; subsequently, the EV suspension underwent sterile filtration under grade A conditions, with a surrounding grade B environment. The entire process was performed in automatic mode, setting the software that manages the entire instrument and regulates its functionality by controlling the weight of permeate and feed bags and controlling pressures in three different flowpath districts (feed, retentate, permeate). The input flow rate was set to 600 mL/minute, and the main valve closure was automatically regulated by the instrument in order to keep the trans membrane pressure at 0.1 bar during the whole filtration procedure.

The full process run was composed of three subsequent phases. In the first phase, the initial volume of CM was recirculated until a 10-fold volume reduction was achieved (first concentration 10×, to about 900 mL); in the second phase the remaining volume (about 900 mL) was washed with 10 volumes of diafiltration buffer (9 L) (NaCl 0.9% added with 0.1% Sucrose); in the third phase, the 900 mL of washed CM was furtherly reduced by half (450 mL) until a total concentration factor of 20× was achieved. The final volume of approximately 450 mL was finally filtered through a sterilizing filter, Supor EKV filter in mini Kleenpak capsules of 0.2 µm (Pall Corporation). An aliquot of concentrated product was used to evaluate EV concentration and size, and then the product was frozen in bags containing EVs resuspended in sodium chloride (0.9%), sucrose (0.1%), and DMSO (1%) [18] at the final concentration of 4 × 10^9^ EVs/mL. Evaluation of the stability of the frozen concentrated product was performed one year after the freezing procedure. The following parameters were evaluated: EV concentration, size distribution, immunophenotype, appearance, sterility, presence of endotoxin, and TEM observation (Supplementary Table S1).

### 2.4. In Process Controls (IPCs)

The following paragraphs describe individually the IPCs performed during the entire production process.

#### 2.4.1. Cell Morphology

Cell morphology was a control aimed to assess the correct behavior of cells during the culture and consisted of a microscopic visual qualitative check to verify the state of adhesion of the cells, carried out by the production personnel using the inverted microscope at a magnification of 40×. The testing was performed during the working phases to monitor the general state of the culture, and the result was recorded in the specific batch record of the production. The adhesion of the cells to the bottom of the flask and their elongated morphology confirmed their correct growth and their viability. The clarity of the medium was an indication of the absence of microbial contamination.

#### 2.4.2. Daily Check of the Confluence

It was a microscopic visual qualitative check, carried out on all flasks, to verify the state of confluency of the cells, to monitor their growth, and the correct time for their subsequent expansion/harvest. It was performed by the production personnel using an inverted microscope at a magnification of 40×. The check was carried out at the same time point of cell morphology to verify the general state of the culture.

#### 2.4.3. Cell Count and Viability

Cell count and viability are critical control parameters aimed at calculating the adequate cell number to seed at a defined cell density. They consisted of the dilution of cells in Trypan Blue (Cytiva HyClone, Uppsala, Sweden) and then the count of viable cells in a Burker’s chamber using the inverted microscope at a 100× magnification.

#### 2.4.4. Immunophenotype

The cytofluorimetric evaluation is aimed at verifying the identity of cells after the stressed step of starvation. The control was carried out on a representative sample of harvested cells post-starvation, analyzing the expression of characteristic surface antigens and markers, such as CD29, CD73, CD105, CD90, CD44, CD14, CD45, CD34 (all from Miltenyi Biotec), and cytoplasmatic albumin (LSBio, Newark, CA, USA). The cytofluorimetric analyses were performed using the Miltenyi Flow Cytometer MACSQuant10 and analyzed with the MACSQuantify software version 2.13 with the 21 CFR Part 11 module, specifically designed to support GMP-compliant workflows.

#### 2.4.5. Microbial Examination

The microbial examination was conducted to demonstrate the absence of contamination in the EV concentrates post-TFF, before the final sterilizing filtration. It was carried out on ≤1% of volume by direct inoculum in the aerobic culture medium (TSB-Tryptone Soya Broth, CPC Biotech, Burago di Molgora, Italy), followed by incubation according to the Ph.Eur. 2.6.13.

#### 2.4.6. Nanoparticle Tracking Analysis (NTA)

The NTA analysis allowed the determination of the number and size of EVs. The control was carried out on a sample of TFF concentrated EVs, as a starting value for the dilution. The NanoSight NS300 device (NanoSight Ltd., Amesbury, UK) was used under steady flow conditions (flow rate = 30). For each EV sample, three 60-s videos were recorded, and the data were processed using the NTA 3.2 software.

#### 2.4.7. Visual Inspection for Visible Particulate Matter

This non-compendial test was aimed at excluding the presence of extraneous particles in the EV bags. Immediately after the filling and the sealing, each bag was visually inspected to verify the absence of visible particulates.

### 2.5. Quality Control Tests on the Final Drug Product

The analytical tests on the drug product (DP), to evaluate its conformity, were carried out in accordance with an internal specification that lists the analyses and methods to be performed for the release of the batch. The acceptance criteria for each test executed are summarized in Table 1.

#### 2.5.1. Particle Size and Concentration Measurement

The NTA was used to determine the size distribution and the concentration of EVs, as reported before in the description of IPCs.

#### 2.5.2. Immunophenotype

The detection and quantification of 37 surface epitopes and of 2 isotypic controls were carried out using the MACSPlex Exosome Kit (Miltenyi Biotec). EVs bound to the Exosome Capture Beads were labelled with allophycocyanin-conjugated exosome detection antibodies targeting universal exosomal markers CD9, CD63, and CD81. The resulting sandwich complexes were analyzed by flow cytometry (Miltenyi Flow Cytometer MACSQuant10 and MACSQuantify software).

#### 2.5.3. Bovine Serum Albumin (BSA) Content Measurement

BSA is a residual contaminant (impurity) derived from the use of FBS in the cell culture medium. The BSA ELISA KIT (Cygnus Technologies, Leland, NC, USA) was used to ensure that the amount of BSA present in DP is lower than the acceptance criteria.

#### 2.5.4. EGF and FGFb Content Measurement

EGF and FGFb are residual contaminants (impurity) derived from the use of these growth factors in the cell culture medium. The Quantikine^®^ Colorimetric hEGF and hFGFb ELISA Kits (R&D Systems, Minneapolis, MN, USA) were used to ensure that the amount of EGF and FGFb present in DP are lower than the acceptance criteria.

#### 2.5.5. Transmission Electron Microscopy (TEM)

The integrity of the EV membranes and the shape of the EVs were assessed by TEM by the company QuTEM (Stockholm, Sweden). Briefly, the specimen was deposited on a hydrophilized TEM grid and blotted off, leaving a thin film of the specimen. A solution of heavy metal salt was then deposited on the support, and the excess was blotted off. The grid was then inserted and observed in the microscope under room temperature conditions. A semi-randomized image acquisition routine was used to ensure unbiased data collection by the microscopists. Once the data were collected, image analyses and reporting were performed using the Vironova Analyzer Software (VAS).

#### 2.5.6. Safety Test

All batches were analyzed for the following safety tests: sterility, endotoxin, and mycoplasma by the company Eurofins Biolab (Vimodrone, Italy) in accordance with European Pharmacopoeia compendial test (Eu. Ph 2.6.27, Eu.Ph 2.6.14, and Eu.Ph 2.6.7, respectively).

#### 2.5.7. Residual Protein Quantification

The Pierce Micro BCA Protein Assay Kit (Thermo Fisher, Waltham, MA, USA) was used to measure the protein (impurity) concentration in the DP. The result of the test was used for data collection to define the acceptance criteria for release. The test result was used both for data collection to define the acceptance criteria for release and for the calculation of EV purity according to the ratio particle concentration/μg of protein.

#### 2.5.8. Appearance

The clarity and the degree of opalescence of the DP is assessed according to Eu.Ph. 2.2.1 and Eu.Ph 2.2.2 on DP bag after thawing. The result must be compliant with the release specifications of the finished product.

#### 2.5.9. Visible Particles

Visible particles in the DP were detected by Eu.Ph 2.9.20 on a sample derived from DP bag after thawing. The result must be compliant with the release specifications of the finished product.

#### 2.5.10. Subvisible Particles

Subvisible particles were assessed according to Eu.Ph. 2.9.19 on a sample derived from DP bag after thawing. The result must be compliant with the release specifications of the finished product.

#### 2.5.11. pH

The pH value was measured according to Eu.Ph 2.2.3 on a sample derived from DP bag after thawing. The result must be compliant with the release specifications of the finished product.

#### 2.5.12. Osmolality

The osmolality of the DP was measured according to Eu.Ph 2.2.35 on a sample derived from DP bag after thawing. The result must be compliant with the release specifications of the finished product.

### 2.6. EV miRNA Content Characterization

Total RNA was isolated from EVs using miRNeasy Serum/Plasma Kit (Qiagen, Valencia, CA, USA). Briefly, 5 × 10^9^ EVs were suspended in 1 mL of Qiazol and RNA was extracted following the manufacturer’s instructions. Total RNA was quantified using the NanoDrop2000 spectrophotometer (Thermo Fisher), and cDNA was synthesized using the miScript II RT Kit (Qiagen, Valencia, CA, USA) following the manufacturer’s instructions. Quantitative real-time (qRT) PCR was performed with miScript SYBR Green PCR kit (Qiagen, Valencia, CA, USA). For miRNA expression analysis, specific oligonucleotide primers (MWG Biotech, Eurofins Scientific, Brussels, Belgium) were used as follows: hsa-miR-222: AGCTACATCTGGCTACTGGGT; hsa-miR-191: CAACGGAATCCCAAAAGCAG; hsa-miR-31: AGGCAAGATGCTGGCATAG; hsa-miR-146a-5p: TGAGAACTGAATTCCATAGGCT; has-miR-29a-3p TAGCACCATCTGAAATCGGTTA; and hsa-miR-24-3p TGGCTCAGTTCAGCAGGA. miRNA expression was normalized using RNU6: CGCAAGGATGACACGCAA.

### 2.7. Experimental In Vitro Model

#### 2.7.1. Cell Culture

Immortalized human foreskin dermal fibroblasts (fHDF/TERT166, Evercyte GmbH, Wien, Austria) were expanded according to the manufacturer’s guidelines. Original vials were thawed and cultured in complete growth media (CGM) (DMEM-F12 with 15 mM HEPES, Evercyte) supplemented with 0.1% G418 (Thermo Fisher) and 10% FBS. Following the first 3 rounds of expansion, a primary cell bank (PCB) of frozen fHDF cells was created by resuspending cells in storage solution and transferring the vials to liquid nitrogen for long-term preservation. From a single vial of PCB, cells were further expanded to create a final working cell bank at the 9th passage (WCB P9), which was stored in liquid nitrogen for future assays. Original cell vials, PCB cells, and WCB cells were tested for the presence of mycoplasma.

#### 2.7.2. Fibrosis Assay

One vial of the WCB P9 was thawed rapidly at 37 °C using a water bath, resuspended in fibroblast CGM, and collected through centrifugation. A total of 10^4^ viable cells/cm^2^ were seeded in a 48-well plate (Falcon, Corning, New York, NY, USA), in 400 μL/well of fresh CGM. The plate was incubated for 24 h at 37 °C and 5% CO_2_ to allow cell attachment. After 24 h, cells were washed with DPBS and media replaced with fibroblast assay medium (FAM) (DMEM-F12 with 15 mM HEPES) supplemented with 0.5% FBS. After overnight incubation, the cell media was replaced either with fresh FAM (negative controls) or FAM supplemented with 10 ng/mL of TGF-β and incubated for 6 h. Thereafter, cells were washed with DPBS, and media were replaced either with fresh FAM (negative and positive controls) or FAM supplemented with different concentrations of EVs (EV-1: 20,000; EV-2: 40,000; and EV-3: 80,000 EVs/cell) and incubated for 24 h before RNA extraction and analysis.

#### 2.7.3. Multiplex qRT-PCR Sample Preparation and Analysis

Samples were prepared and analyzed by Cells-to-Ct 1-Step TaqMan Kit (Thermo Fisher), following the manufacturer’s instructions. Briefly, post-treatment cells were lysed directly on the plate with the provided Lysis Solution, supplemented with DNAse to improve RNA purity. After the addition of Stop Solution, whole RNA samples could be stored at −80 °C for up to one month before analysis. Following RNA extraction, 2 μL of the extract was used as a template for multiplex qRT-PCR. Each sample was analyzed in triplicate. For each well of a 96-well reaction plate, the following reagents were mixed: 5 μL of TaqMan 1-Step qRT-PCR Mix, 1 μL of Primer 1 (GAPDH—FAM), 1 μL of primers, and 11 μL of molecular grade H_2_O.

Primers used were pre-built and provided by Thermo Fisher as per the following specifications:

GAPDH (Hs99999905_m1): Chr.10: 88935074-88991397 on Build GRCh38. Dye: FAM

ACTA 2 (Hs05005341_m1): Chr.12: 6534405-6538375 on Build GRCh38. Dye: VIC.

The thermocycler conditions were set as follows: pre-cycle at 50 °C for 5 min, ramp-up at 95 °C for 20 s, followed by 40 cycles of 95 °C for 3 s and 60 °C for 30 s. Finally, relative quantification (RQ) of gene expression was calculated using the ΔΔCt method, with respect to the positive control.

#### 2.7.4. In Vitro Model on Activated Hepatic Stellate Cells

Human hepatic stellate cells LX-2 (Sigma Aldrich, St. Louis, MO, USA) were maintained in DMEM high glucose (4.5 g/L, Euroclone, Pero, MI, Italy) supplemented with 2% Fetal Calf Serum and 2 nM L-Glutamine (Lonza) and used until passage 6. All cells were maintained in a humidified 5% CO2 incubator at 37 °C. LX-2 were seeded at a density of 15,000 cells/cm^2^, synchronized over-night in serum-deprived medium, and incubated with 10 ng/mL of TGF-β for 6 h to induce activation. Thereafter, cells were incubated for 24 h with 50,000 EVs/cells before RNA extraction and analysis, as previously reported [13].

### 2.8. Experimental In Vivo Model

Six-week-old male NOD.CB17-Prkdcsicd/J mice were purchased from ENVIGO (S. Pietro al Natisone, Udine, Italy). The development of liver fibrosis was induced through intraperitoneal injection of thioacetamide (TAA, 200 mg/kg, Sigma-Aldrich Corporation, St. Louis, MO, USA) twice a week, as described [16,17]. A preliminary experiment was conducted to determine the optimal timing for initiating EV treatment based on the onset of hepatic fibrosis. Four groups of mice (n = 6 per group) were used in this preliminary setup phase. Animals were sacrificed after 2, 4, 6, and 8 weeks of TAA administration. At each time point, histological and functional analyses were performed on liver tissue and plasma samples.

To evaluate the effect of EVs, 4 weeks after the beginning of TAA administration, TAA mice were randomly divided and intravenously injected with the vehicle alone (n = 8) or with EVs (5 × 10^9^, n = 8) [12], once a week for 4 weeks. The dose of EVs was selected based on results previously obtained in vivo using non-GMP-grade EVs [12]. The end of the experiment was set 8 weeks after the beginning of TAA administration, and blood and liver were recovered for biochemical, histological, and molecular analyses.

#### 2.8.1. Liver Function Analyses

Liver function tests, including albumin, total protein, and transaminases (ALT), were performed on plasma samples at the Clinical Biochemistry Laboratory of Città della Salute e della Scienza using the AU5800 analyzer (Beckman Coulter, Brea, CA, USA). ALT was measured using an enzymatic method in accordance with the International Federation of Clinical Chemistry recommendations, while albumin and total proteins were assessed via standard colorimetric assays. Results are expressed in g/dL for albumin and total proteins, and U/L for ALT.

#### 2.8.2. Histological Analyses

Liver morphology was evaluated through formalin-fixed paraffin-embedded tissue staining. Paraffin kidney sections (5 μm thick) were stained for microscopic evaluation with hematoxylin–eosin (Sigma-Aldrich) or Sirius Red (Bio-Optica, Milano, Italy). In liver sections stained with Sirius Red, fibrosis was quantified by measuring collagenous fibrotic areas stained in red, as previously described [12]. Briefly, red staining was quantified in 10 random fields per section from images taken at a magnification of 400×, using multiphase image analyses with the ImageJ software v1.49s.

#### 2.8.3. Molecular Analyses

Hepatic tissue was preserved in RNAlater Stabilization Solution (Thermo Fisher) for molecular analyses. Three different small pieces of liver tissue were recovered from every animal. Total RNA was extracted from liver tissues. Briefly, mouse hepatic tissue was suspended in 500 µL of TRIzolTM solution (Ambion, Thermofisher) in microcentrifuge tubes and homogenized in a Bullet blender (Next Advance Inc., Troy, NY, USA) at a speed of 8 rpm for 3 min using 3.2 mm size zirconium beads. An amount of 500 µL of TRIzolTM solution was added to the liquid phase, and the tubes were centrifuged at 13,000× *g* for 10 min at 4 °C. Supernatant was transferred to clean tubes and subjected to RNA isolation. Total RNA was quantified using the NanoDrop2000 spectrophotometer (Thermo Fisher) and either used immediately or stored at −80 °C until further use. cDNA was synthesized by retrotranscribing 400 ng of total RNA using the High-Capacity cDNA reverse transcription kit (Thermo Fisher, Waltham, MA, USA), according to the manufacturer’s protocol. qRT PCR was performed with Power SYBR Green PCR Master Mix (Thermo Fisher, Waltham, MA, USA) and specific oligonucleotide primers (MWG Biotech, Eurofins Scientific, Brussels, Belgium) as follows: m-GAPDH (Forward: TGTCAAGCTCATTTCCTGGTA; Reverse: TCTTACTCCTTGGAGGCCATGT), m-Alpha-SMA (Forward: CATCTCCGAAGTCCAGCACA; Reverse: GACGCACCACTGAACCCTAA), m-COL1A1 (Forward: ACCTTGTTTGCCAGGTTCAC; Reverse: ATCTCCCTGGTGCTGATGGAC), m-IL-1beta (Forward: GAAATGCCACCTTTTGACAGTG; Reverse: TGGATGCTCTCATCAGGACAG), and m-IFNgamma (Forward: TAGCCAAGACTGTGATTGCGG; Reverse: AGACATCTCCTCCCATCAGCAG).

### 2.9. Statistical Analyses

Statistical analysis was performed using the GraphPad Prism software version 8.0 (GraphPad Software, Inc., La Jolla, CA, USA). All data are shown as mean ± SD. For in vitro and in vivo experimental procedures, comparison among control groups and the different experimental groups was performed using a one-way ANOVA, followed by Tukey’s or Dunnett’s multiple comparison test. A *p*-value < 0.05 was considered significant.

## 3. Results

### 3.1. EV Manufacturing

As recommended by the European Commission (Guidelines on Good Manufacturing Practice specific to Advanced Therapy Medicinal Products), a MCB was produced from a single donor and certified for the validation of an allogeneic product (HLSC-EVs), which do not require a match between the donor and the patient [18]. Three different EV batches were produced under GMP-compliant conditions in a facility authorized for production of Advanced Therapy Medical Products (ATMPs) (GMP certification number: aM81/2025).

Each EV batch was obtained as follows: Two vials of the same CS were seeded and expanded to obtain 16 HFs. When the cells reached confluency, the HFs were subjected to starvation. Cytofluorimetric analyses indicated that HLSCs, after an overnight serum deprivation, maintained a stable phenotype (Supplementary Figure S1) comparable to cells cultured in the expansion medium [11,12]. Specifically, they expressed MSC markers (CD90, CD29, CD73, and CD105) and did not express hematopoietic markers (CD14, CD45, and CD34). The following day, the CM was pooled and immediately subjected to TFF. The final TFF concentrate was filtered and diluted in freezing solution to achieve a final concentration of 4 × 10^9^ EVs/mL, and then frozen in bags containing approximately 50 mL of EV suspension (Figure 1). Table 2 summarizes the main culture and isolation conditions for each EV batch.

### 3.2. Quality Control Tests on Drug Product

Quality control analyses of the DP demonstrated that the different batches were homogeneous in terms of EV concentration (EVs/mL), size distribution, and phenotype, as well as for physicochemical parameters such as pH, osmolality, and purity indices, including the absence of visible and subvisible particles and a low protein content. As reported in Table 3, the results show overlapping characteristics across the three different EV preparations, demonstrating that the GMP production process is both consistent and reproducible. Moreover, the results obtained from the stability study suggest that the same characteristics were maintained at least one year after the freezing (Supplementary Table S1).

**Figure 2:**
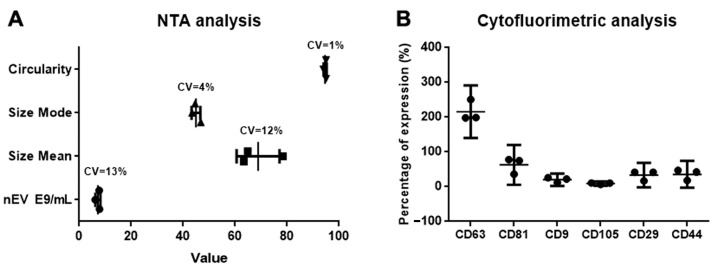
Graphic representations of the distribution and the statistical analysis of NTA (**A**) and cytofluorometric (**B**) parameters among the three EV batches. (**A**) Results are shown as a single value + SD of three independent measurements, and the coefficient of variation (CV) among them was calculated. (**B**) Results are shown as a single value + SD of three independent results with *p* < 0.972.

Statistical analysis of the cytofluorimetric characterization parameters and particle distribution data revealed that the three batches were statistically equivalent, with a coefficient of variation below 15% for EV size and concentration, and a *p*-value of 0.972 for EV marker expression (Figure 2). The presence of contaminants fulfilled the release criteria, and sterility tests confirmed the absence of bacteria, mycoplasma, or endotoxins (Table 3).

To assess EV purity, we quantified the residual protein content and calculated the particle-to-protein ratio (particle concentration per μg of protein). According to the criteria proposed by Webber and Clayton, ratios >3 × 10^10^ particles/μg indicate high purity, whereas ratios <1.5 × 10^9^ particles/μg suggest impure preparations [19]. As shown in Table 3, all three GMP-grade batches exhibited ratios ranging from 5 × 10^9^ to 3.9 × 10^10^ particles/μg, demonstrating that the GMP-grade HLSC-EVs meet the purity standards established by Webber and Clayton.

**Figure 3:**
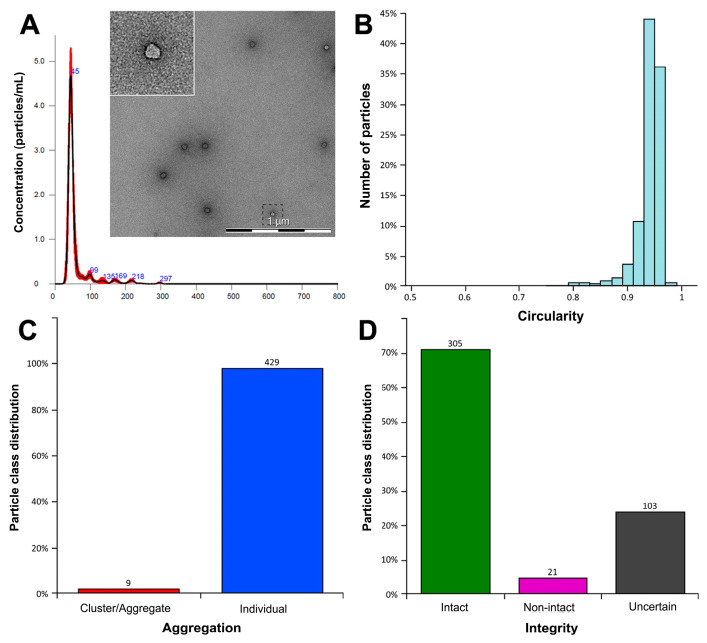
Characterization of EVs. (**A**) Representative graph illustrating EV size distribution via NTA (red lines indicate the mean of the three recorded videos), alongside a representative micrograph of TEM showing intact EVs. (**B**) Circularity analysis: particles with circularity values between 0.5 and 1 are displayed in the histogram. (**C**) Histogram analysis showing the quantification of the detected individual particles overlaid with blue outlines and particle clusters/aggregates overlaid with red outlines. They were classified according to the following criteria: Cluster/aggregate (red) containing at least two particles; individual (blue) is an isolated particle. (**D**) Histogram analysis showing the integrity classification of the EVs. The particles were classified according to the following criteria: intact (green) with a homogeneous density and well-defined exterior (Size > 40 nm); non-intact (purple) with a heterogeneous density or faint exterior (Size > 40 nm); uncertain (black) with a size < 40 nm and particles that cannot ambiguously defined as “Intact” or “Non-Intact”.

TEM analysis showed a low concentration of evenly distributed EVs. The majority of the particles appeared as bright and circular structures, characteristic of intact EVs. Occasionally, irregularly shaped particles, indicative of non-intact EVs, were observed. The EVs predominantly appeared as single entities, although sporadic clusters and small debris were occasionally detected (Figure 3).

### 3.3. EV miRNA Content

The presence of six miRNAs, previously reported to be expressed in non-GMP HLSC-EVs produced by ultracentrifugation [20], was evaluated in the DP. All six miRNAs were confirmed in EVs manufactured under GMP conditions using TFF (Table 4), with expression levels comparable to those observed in non-GMP-EVs (Supplementary Table S2).

### 3.4. In Vitro Functional Test

**Figure 4:**
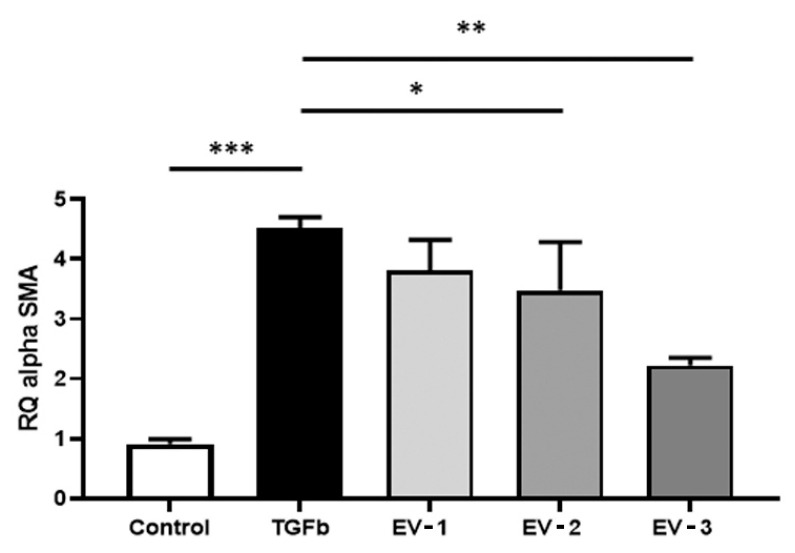
Evaluation of dose–response EV effect on activated fibroblasts. qRT-PCR analysis of the expression of a-SMA in activated fibroblasts after 24 h of incubation with different concentrations of EVs (EV-1: 20,000 EVs/cell, EV-2: 40,000 EVs/cell, EV-3: 80,000 EVs/cell). Fibroblasts activated with TGF-β (10 ng/mL), not incubated with EVs, were used as the reference control (TGFb). Fibroblasts cultured without TGF-β were used as the negative control (control). Gene expression levels were normalized to those of the housekeeping gene GAPDH. Results are shown as mean ± SD of four independent experiments performed in triplicate. * *p* ≤ 0.01, ** *p* ≤ 0.001, and *** *p* ≤ 0.0001.

To evaluate the effect of EVs on the activated phenotype of fibroblasts, TGF-β-activated cells were incubated with different concentrations of EVs (Figure 4). Treatment with EVs led to a reduction in the expression of alpha smooth muscle actin (alpha-SMA), a key marker of fibroblast activation, after 24 h of incubation. Specifically, the reduction in alpha-SMA expression was significant in fibroblasts treated with the higher concentrations of EVs (EV-2 and EV-3, corresponding to 40,000 and 80,000 EVs/cell, respectively). The in vitro antifibrotic effect was confirmed also on the hepatic stellate cells (Supplementary Figure S2).

### 3.5. In Vivo Efficacy

**Figure 5:**
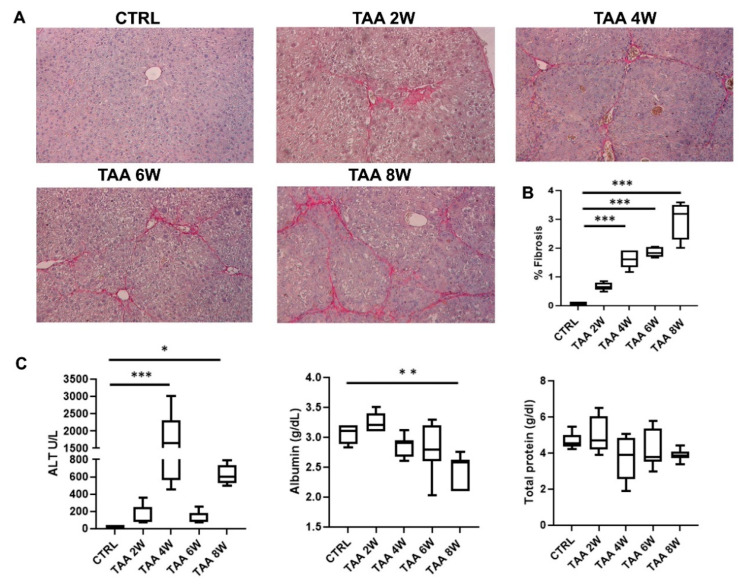
Experimental setup to determine the onset of liver fibrosis. (**A**) Representative light micrographs of Sirius Red-stained liver sections from a healthy control mouse (CTRL) and from TAA-treated mice sacrificed at different time points during TAA administration: week 2 (TAA 2W), week 4 (TAA 4W), week 6 (TAA 6W), and week 8 (TAA 8W). Red staining indicates collagen fibers, used as a marker of liver fibrosis. (**B**) Quantification of fibrosis in healthy control mice (CTRL, n = 8) and in TAA-treated mice sacrificed at weeks 2, 4, 6, and 8 (n = 6 per group), performed by multiphase image analysis of 10 randomly selected fields per section (original magnification, 400×). Data are presented as mean ± SD. Statistical analysis was performed using a one-way ANOVA. *** *p* < 0.0001. (**C**) Evaluation of plasma levels of ALT, albumin, and total proteins in healthy control mice (CTRL, n = 8) and in TAA-treated mice sacrificed at weeks 2, 4, 6, and 8 (n = 6/group). Results are shown as mean ± SD. * *p* ≤ 0.05, ** *p* ≤ 0.0005, and *** *p* ≤ 0.0001.

To evaluate the therapeutic potential of EVs in hepatic fibrosis, we induced chronic liver injury with TAA treatment. The experimental setup revealed a marked increase in liver fibrosis in TAA-treated mice as early as week 4, with further progression by week 8, as evidenced by Sirius Red staining (Figure 5A,B). Biochemical analyses confirmed that TAA administration resulted in impaired hepatic function (Figure 5C). Plasma ALT levels were significantly elevated at week 4, reflecting acute hepatocellular injury due to TAA accumulation. A transient decrease in ALT was observed at week 6, followed by a further significant increase at week 8, corresponding to the histological establishment of hepatic fibrosis and indicating the transition to chronic liver injury. At week 8, chronic hepatic damage was also supported by a reduction in plasma albumin and total protein levels in TAA-treated mice (Figure 5C). 

**Figure 6:**
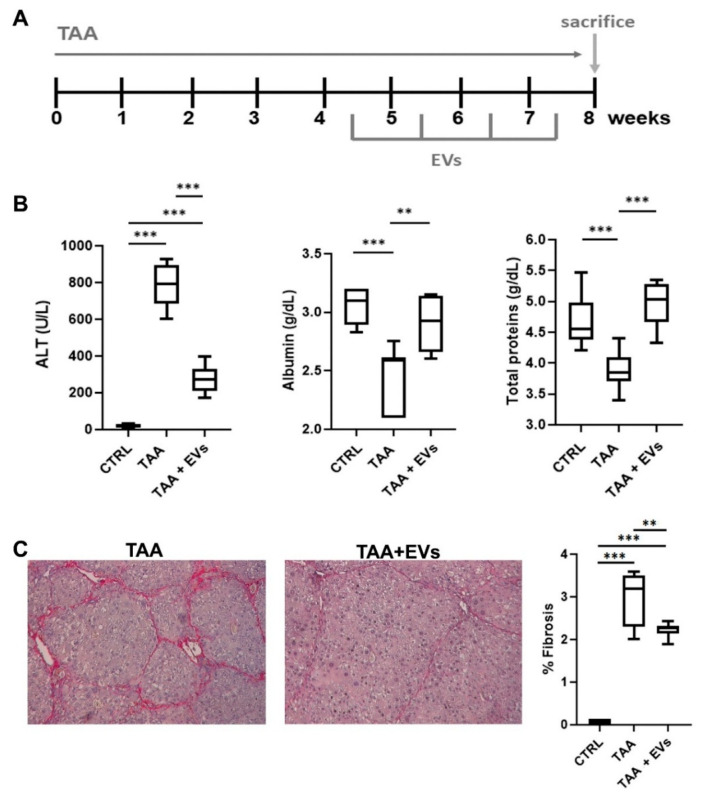
Effect of EV treatments on hepatic function. (**A**) Schematic representation of the experimental setting to test EVs in TAA mice, showing the weeks of TAA administrations, of EV treatments (5 × 10^9^ EVs/injection), and of sacrifice. (**B**) Evaluation of the plasma levels of ALT, albumin, and total proteins of control healthy mice (CTRL, n = 8) and TAA mice sacrificed at week 8, treated or not with EVs (TAA 8W, TAA 8W + EVs, n = 8). Results are shown as mean ± SD. ** *p* ≤ 0.001 and *** *p* ≤ 0.0001. (**C**) Representative light microscopy micrographs of Sirius Red-stained liver sections and histological quantification of fibrosis in healthy control mice (CTRL, n = 8), in TAA mice sacrificed at week 8 injected with EVs (TAA + EVs, n = 8), or with vehicle alone (TAA, n = 8) by multiphase image analyses of 10 fields per section (original magnification 400×). The data shown represents mean ± SD. ** *p* ≤ 0.006 and *** *p* ≤ 0.0001.

EV treatment started at week 4 during TAA administration, when histological signs of hepatic fibrosis development were already evident, and was delivered intravenously once a week for 4 weeks (Figure 6A). EV injections significantly reduced ALT levels and increased albumin and total protein concentration in plasma from TAA-treated mice sacrificed at week 8 (Figure 6B). Histological analyses confirmed the therapeutic effect, as EV treatments significantly reduced fibrosis in TAA mice sacrificed at week 8 (Figure 6C). These findings suggest that EV treatments effectively improve liver function and morphology in the TAA-induced chronic liver disease model by limiting fibrosis progression.

**Figure 7:**
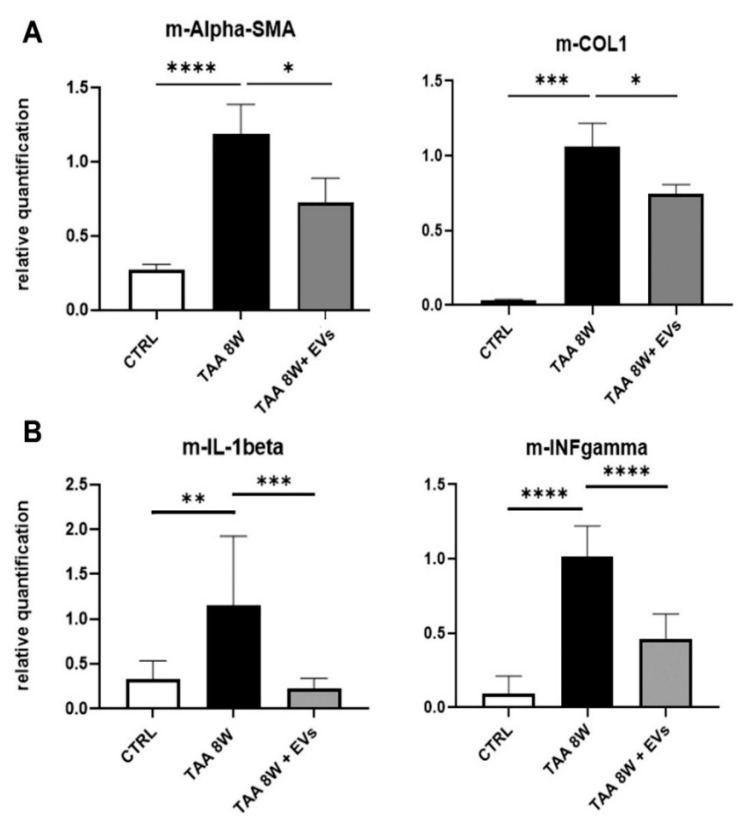
Effect of EVs on molecular markers of fibrosis and inflammation in vivo. Gene expression levels of fibrotic markers (Col I and alpha-SMA) (**A**) and of pro-inflammatory cytokines (IL-1β and IFN-γ) (**B**) in livers of healthy mice (CTRL, n = 8) and of TAA-mice injected with EVs (TAA 8W + EVs, n = 6) or with vehicle alone (TAA 8W, n = 8). Data are expressed as relative quantification using the ΔΔCt method. Normalization was made using m-GAPDH as a housekeeping gene. * *p* ≤ 0.03, ** *p* < 0.05, *** *p* ≤ 0.0002, and **** *p* < 0.0001.

Furthermore, mRNA expression levels of key fibrosis markers, such as Collagen I (Col I) and alpha-SMA, were analyzed in liver samples. Their gene expression levels were reduced following EV administrations, supporting the antifibrotic effects of EVs (Figure 7). Molecular analyses also showed that EV treatments significantly modulated the expression of genes involved in inflammation. In particular, TAA-treated mice that received EVs exhibited a significant reduction in pro-inflammatory cytokines, such as interleukin-1 beta (IL-1β) and interferon gamma (IFN-γ) (Figure 7).

## 4. Discussion

EVs have recently garnered significant attention as promising candidates for cell-free therapeutic applications across various pathologies. EV-based therapeutics derived from native MSCs are classified as biological medicinal products under the regulatory frameworks in Europe, the USA, Australia, and Japan. A biological medicinal product contains one or more active substances produced by, or derived from, living cells [21]. One of the primary challenges in developing EV-based therapies is optimizing large-scale production while ensuring high yield and batch-to-batch consistency. Additionally, manufacturing any biological therapeutic intended for human use must comply with GMP requirements, detailed in Volume 4 of EudraLex [17], and adhere to Commission Directive 2003/94/EC on the principles of GMP for investigational medicinal products for human use, such as the guidelines of the International Council for Harmonization (ICH). Manufacturers are required to ensure the quality of the finished product through the adequate characterization of critical components, stability, purity, and strength according to ICHQ5D. Compliance with GMP is required at all stages of EV production, including the cell culture system (to minimize contamination risk), EV purification, characterization, quality control testing, and thorough documentation of all procedures [21,22,23,24,25]. Addressing these challenges is critical for translating EVs into viable therapeutic products.

This study aimed to demonstrate the feasibility, consistency, and biological activity of GMP-produced HLSC-EVs, which represent critical prerequisites for clinical translation. We investigated HLSCs as a scalable and clinically relevant source for therapeutic EVs. HLSCs have already been tested in a Phase I clinical trial and shown to be safe [11], further supports their translational potential. Compared to cell-based therapies, EVs offer multiple advantages, including enhanced stability through freeze–thaw cycles, the possibility to sterilize the final product by filtration, and reduced immunogenicity [26].

We successfully produced three independent batches, derived from the same starting material, of HLSC-EVs under GMP-compliant conditions within a certified facility authorized for ATMP production. Quality control analyses confirmed consistency in EV yield, size distribution, and surface marker expression across batches. TEM further revealed that the majority of EVs retained their structural integrity, with only a small proportion of non-intact vesicles, confirming the robustness of the GMP production process. EV batches were produced using TFF, a scalable purification method for clinical-grade EVs. The use of TFF is consistent with preclinical and clinical studies that employed GMP-compatible TFF protocols to isolate EVs from umbilical cord derived MSCs, which were subsequently applied in a rat model of bronchopulmonary dysplasia [27], as well as in first-in-human intracochlear applications and in tethered spinal cord release surgery [28,29].

To evaluate the biological activity of GMP-grade HLSC-EVs, we employed an in vitro assay targeting activated fibroblasts. Our results show that the EVs were capable of attenuating their activated phenotype, suggesting an antifibrotic effect. We further assessed the therapeutic efficacy of GMP-grade HLSC-EVs in vivo using the TAA-induced murine model of liver fibrosis, a well-established and widely used approach to study chronic liver injury. Chronic TAA exposure induces hepatocellular damage, oxidative stress, and inflammation, leading to hepatic stellate cell activation and excessive extracellular matrix deposition—processes that closely mirror human liver fibrosis. This model has also been employed in studies demonstrating the antifibrotic activity of EVs derived from Wharton’s jelly MSCs and adipose tissue stromal vascular fractions [16,17], underscoring its relevance to preclinical testing.

Treatment with HLSC-EVs significantly reduced liver fibrosis at both histological and molecular levels and improved liver function. These findings are consistent with previous studies using non-GMP HLSC-EVs; notably, EVs isolated by ultracentrifugation and tested in a methionine- and choline-deficient diet-induced model of liver fibrosis exhibited comparable therapeutic effects [12]. Collectively, these results indicate that the biological activity of HLSC-EVs is preserved following adaptation to GMP production protocols and that the transition from research-grade to clinical-grade manufacturing does not compromise therapeutic efficacy.

Although the antifibrotic effects exerted by HLSC-derived EVs has been demonstrated both in vitro and in vivo, the precise mechanism of action (MoA) behind HLSC-EVs in fibrosis reversal was not investigated in this study. The MoA of new therapeutic products does not necessarily need to be elucidated prior to first-in-human studies, as long as scientific data on expected biological activity is adequate. However, MoA data are expected to be detailed over the course of Phase I to III studies. Previous reports have indicated that HLSC-derived EVs exert antifibrotic effects through the delivery of bioactive cargo, including microRNAs, mRNAs, and proteins that modulate inflammatory responses, and fibrogenic pathways [10,12]. Further elucidation of the MoA will enhance the development of HLSC-EVs as a medicinal product. Mechanistic studies remain an important future direction, and further investigation into the EV cargo and its interaction with recipient cells would help to clarify the MoA.

This study has several limitations to be acknowledged. First, the antifibrotic efficacy was tested in a single animal model of liver fibrosis, which may not fully recapitulate all forms of chronic liver disease. In addition, dose–response, biodistribution studies, and long-term outcomes following EV treatment were not evaluated; therefore, the durability and persistence of therapeutic effects remain to be assessed. Finally, although batch consistency was confirmed under GMP conditions, inter-donor variability of HLSCs was not investigated, which may influence EV composition and efficacy in future clinical applications.

## 5. Conclusions

Taken together, this study demonstrates the feasibility of producing HLSC-EVs under fully GMP-compliant conditions using a standardized and scalable manufacturing workflow. This ensures consistent physicochemical and biological properties that support their development as a clinical-grade biological product. Notably, these GMP-grade EVs retained antifibrotic activity both in vitro and in vivo, confirming that the transition from research-grade isolation procedures to GMP-compatible production does not compromise their therapeutic potential.

Importantly, the manufacturing strategy described here integrates GMP-compliant cell expansion, scalable TFF purification, and rigorous quality control testing, thereby providing a practical framework for the production of clinical-grade EVs intended for therapeutic use.

The use of a well-characterized stem cell source together with a reproducible and potentially scalable manufacturing protocol represents a critical step toward the standardization of EV-based therapeutics, which remains one of the major challenges in the field. Although further studies will be required to better define the mechanism of action, optimize dosing strategies, and evaluate long-term safety and biodistribution, the results presented in this paper provide strong preclinical evidence supporting the advancement of GMP-produced HLSC-EVs toward early-phase clinical trials.

Overall, this work establishes a translational platform for the development of HLSC-EVs as a novel cell-free therapeutic strategy for liver fibrosis and potentially other fibrotic diseases.

## Figures and Tables

**Table 1:** Acceptance criteria for EV batch release.

Test	Acceptance Criteria
**Appearance (Clarity and degree of opalescence)**	Not more opalescent than the reference solution
**Visible particles**	Essentially free from visible particles
**Subvisible particles**	≥10 μm: ≤6000 particles per container ≥25 μm: ≤600 particles per container
**EV concentration**	≥3.5 × 10^9^ EV/mL
**Immunophenotype (identity)**	Positive for CD63, CD81, CD9, CD29, CD44, CD105
**Size distribution**	60–150 nm mean vesicle size 40–100 nm mode size
**BSA residual content**	≤5 ng/mL
**EGF residual content**	≤100 pg/mL
**FGFb residual content**	≤100 pg/mL
**pH**	5.5–7.5
**Osmolality**	<850 mOsmol/kg
**Sterility**	Culture negative
**Endotoxin**	≤1 EU/mL
**Mycoplasma**	Not detected

EV: extracellular vesicle; BSA: bovine serum albumin; EGF: epidermal growth factor; FGF-b: basic fibroblast growth factor.

**Table 2:** Summary of the three EV batches’ production process.

	Batch		
	nEV HLSC-CLF-05/23	nEV HLSC-CLF-06/23	nEV HLSC-CLF-07/23
**Length of process (days)**	15	15	15
**Length of starvation (hours:minutes)**	18:00	17:30	18:00
**Volume of CM (mL)**	8848	8890	8233
**Volume of concentrated product after TFF (mL)**	399	532	424

CM: conditioned medium; TFF: tangential flow filtration.

**Table 3:** Result of quality control tests on DP.

	Batch		
Test	nEV HLSC-CLF-05/23	nEV HLSC-CLF-06/23	nEV HLSC-CLF-07/23
**EV concentration**	6.1 × 10^9^ EV/mL	7.8 × 10^9^ EV/mL	7.7 × 10^9^ EV/mL
**EVs/batch**	2.43 × 10^12^	4.15 × 10^12^	3.26 × 10^12^
**EVs/HF**	1.51 × 10^11^	2.59 × 10^11^	2.04 × 10^11^
**EVs/cm^2^**	8.83 × 10^7^	1.51 × 10^8^	1.15 × 10^8^
**Size distribution**	Mean = 78.4 nm Mode = 46.8 nm	Mean = 63.4 nm Mode = 43.5 nm	Mean = 64.9 nm Mode = 44.7 nm
**Immunophenotype**	CD63 = 251.1% CD81 = 36.6% CD9 = 12.3% CD105 = 6.4% CD29 = 17.5% CD44 = 18.4%	CD63 = 199.2% CD81 = 78.0% CD9 = 22.9% CD105 = 11.1% CD29 = 42.0% CD44 = 42.6%	CD63 = 198.3% CD81 = 75.4% CD9 = 26.3% CD105 = 10.6% CD29 = 42.1% CD44 = 47.5%
**Appearance**	Absence of extraneous particles	Absence of extraneous particles	Absence of extraneous particles
**Visible particles**	Free of visible particles	Free of visible particles	Free of visible particles
**Subvisible particles**	≥10 μm = 283 ≥25 μm = 0	≥10 μm = 518 ≥25 μm = 6	≥10 μm = 367 ≥25 μm = 3
**pH**	5.6	5.6	5.6
**Osmolality**	376 mOsmol/kg	444 mOsmol/kg	436 mOsmol/kg
**EGF residual content**	<3.91 pg/mL	<3.91 pg/mL	<3.91 pg/mL
**FGFb residual content**	<10 pg/mL	<10 pg/mL	<10 pg/mL
**BSA residual content**	0.54 ng/mL	1.15 ng/mL	0.96 ng/mL
**Residual protein content**	1.3 µg/mL	0.2 µg/mL	0.2 µg/mL
**Particles/μg protein**	0.47 × 10^10^	3.91 × 10^10^	3.83 × 10^10^
**Sterility**	Culture negative at LOD	Culture negative at LOD	Culture negative at LOD
**Mycoplasma**	Not detected	Not detected	Not detected
**Endotoxin**	<0.0500 EU/mL	<0.0500 EU/mL	<0.0500 EU/mL
**TEM**	Intact membrane	Intact membrane	Intact membrane

EV: extracellular vesicle; BSA: bovine serum albumin; EGF: epidermal growth factor; TEM: transmission electron microscopy; LOD: limit of detection.

**Table 4:** EV miRNA content.

DP		
miRNA	CT Mean	SD
hsa-miR-222	27.187	0.936
hsa-miR-24-3p	28.211	0.928
hsa-miR-29a-3p	29.616	0.848
hsa-miR-31-5p	30.202	1.003
hsa-miR-191	30.719	0.812
hsa-miR-146a-5p	32.068	1.373

## Data Availability

The data that support the findings of the present study are available from the corresponding author upon reasonable request.

## Supplementary Materials

The following supporting information can be downloaded at: https://www.mdpi.com/article/10.3390/cells15080661/s1, Figure S1: Representative FACS analyses of HLSCs; Table S1: Result of quality control tests on DP for stability study; Figure S2: Evaluation of EV effect on activated hepatic stellate cells; Table S2: EV miRNA content.

## Abbreviations

The following abbreviations are used in this manuscript:

HLSCs	Human Liver Stem Cells
MSCs	Mesenchymal Stem Cells
EVs	Extracellular Vesicles
GMP	Good Manufacturing Practice
ECM	Extracellular Matrix
TGF	Transforming Growth Factor
TAA	Thioacetamide
TFF	Tangential Flow Filtration
MCB	Master Cell Bank
CS	Cell Stock
HFs	Hyperflasks
IPC	In Process Control
TEM	Transmission Electron Microscopy
ATMP	Advanced Medical Product
BSA	Bovine Serum Albumin
EGF	Epidermal Growth Factor
FGF	Fibroblast Growth Factor
SMA	Smooth Muscle Actin
IL-1	Interleukin 1
IFN	Interferon
ALT	Alanine Aminotransferase
