# Supplementary material for: Good Manufacturing Practice-Derived Human Liver Stem Cell Extracellular Vesicles Attenuate Liver Fibrosis In Vivo
Source: Cells. 2026 Apr 9;15(8):661. doi: 10.3390/cells15080661 (PMC13114769; doi:10.3390/cells15080661)
Supplement: Supplementary file 1 [file cells-15-00661-s001.zip › cells-4180986-supplementary.pdf]

Supplementary Materials

Good manufacturing practice-derived human liver stem cell extracellular vesicles attenuate *in vivo* liver fibrosis

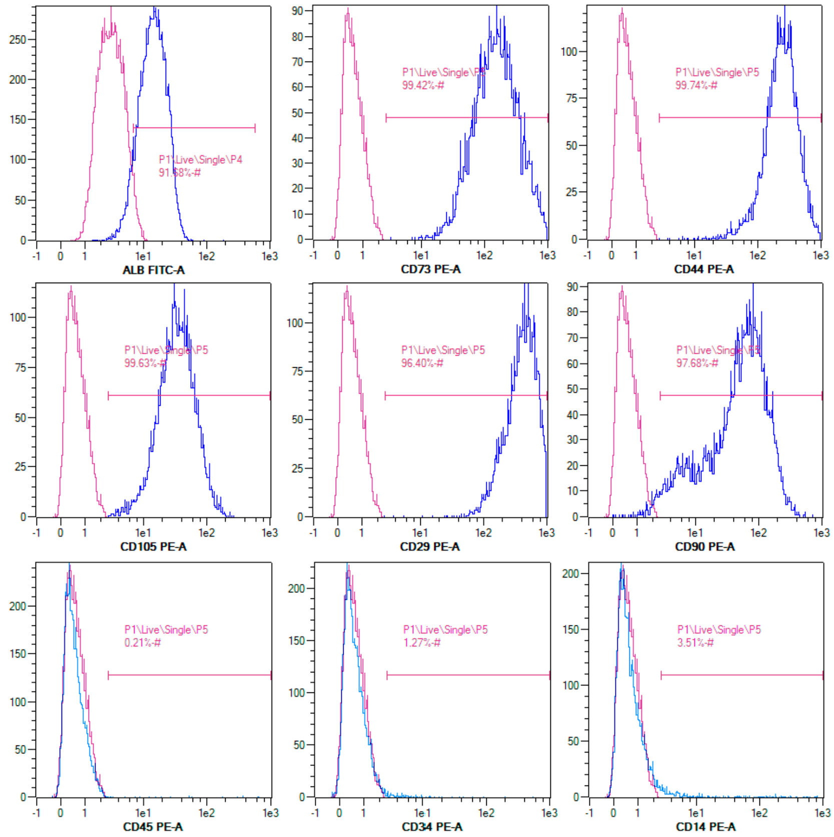

Supplementary Figure 1. Representative FACS analyses of HLSCs, after overnight starvation, showing the expression of albumin (ALB), CD73, CD44, CD105, CD29, and CD90 (blue histograms). CD45, CD34 and CD14 are not expressed. Pink histograms represent isotypic control.

Supplementary Table 1. Result of quality control tests on DP for stability study.

		Batch		
**Test**	**Acceptance criteria**	**nEV HLSC-CLF-05/23**	**nEV HLSC-CLF-06/23**	**nEV HLSC-CLF-07/23**
**EV concentration**	≥ 3.5 x10^9 EV/mL	5.2 x10^9 EV/mL	3.6 x10^9 EV/mL	3.8 x10^9 EV/mL
**Size distribution**	60-150 nm mean size<br>40-100 nm mode size	Mean = 71.1 nm<br>Mode = 44.6 nm	Mean = 86.0 nm<br>Mode = 48.8 nm	Mean = 96.1 nm<br>Mode = 51.8 nm
**Immunophenotype**	Positive for CD63, CD81, CD9, CD29, CD44, CD105	CD63= 238.7%<br>CD81= 46.4%<br>CD9= 15.5%<br>CD105 = 7.3%<br>CD29 = 23.7%<br>CD44 = 24.7%	CD63 = 230.4%<br>CD81 = 19.4%<br>CD9 = 16.0%<br>CD105 = 10.2%<br>CD29 = 32.8%<br>CD44 = 43.5%	CD63=187.8%<br>CD81=86.4%<br>CD9=25.8%<br>CD105=9.3%<br>CD29= 42.5%<br>CD44=58.3%
**Appearance**	Absence of extraneous particles	Absence of extraneous particles	Absence of extraneous particles	Absence of extraneous particles
**Sterility**	Culture negative	Culture negative at LOD	Culture negative at LOD	Culture negative at LOD
**Endotoxin**	< 0.5 EU/mL	< 0.0500 EU/mL	< 0.0500 EU/mL	< 0.0500 EU/mL
**TEM**	Intact membrane	Intact membrane	Intact membrane	Intact membrane

Supplementary Table 2. EV miRNA content.

non-GMP HLSC-EVs		
**miRNA**	**CT mean**	**SD**
hsa-miR-222	27.138	2.296
hsa-miR-24-3p	27.358	2.366
hsa-miR-31-5p	28.587	2.454
hsa-miR-29a-3p	28.763	2.857
hsa-miR-191	30.537	2.225
hsa-miR-146a-5p	31.912	2.922

Data are expressed as mean CT value ± SD (n = 3).

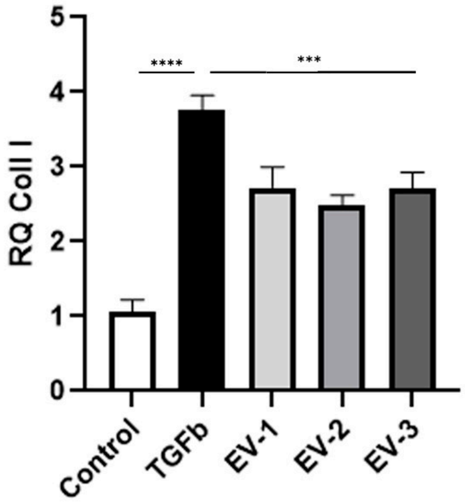

Supplementary Figure 2. Evaluation of EV effect on activated hepatic stellate cells. qRT-PCR analysis of the expression of Collagen I (Coll I) in activated LX-2 cells after 24 hours of incubation with EVs (50,000 EVs/cell). LX-2 cells activated with TGF-β (10 ng/mL), not incubated with EVs, were used as reference control (TGFb). LX-2 cells cultured without TGF-β were used as negative control (control). Gene expression levels were normalized to those of the housekeeping gene TBP. Results are shown as mean ± SD of three independent experiments performed in triplicate. ***$p \leq 0.001$ and ****$p \leq 0.0001$.
